# Functional cellulose-based hydrogels as extracellular matrices for tissue engineering

**DOI:** 10.1186/s13036-019-0177-0

**Published:** 2019-06-20

**Authors:** Sayan Deb Dutta, Dinesh K. Patel, Ki-Taek Lim

**Affiliations:** 1 Biorobotics Laboratory, Department of Biosystems Engineering, Kangwon National University, Chuncheon, Republic of Korea; 2 The Institute of Forest Science, Kangwon National University, Chuncheon, 24341 Republic of Korea

**Keywords:** Cellulose, Hydrogels, Scaffolds, Extracellular matrices, Tissue engineering

## Abstract

Cellulose-based hydrogels are immensely important for tissue engineering. In this review, we attempt to document the source, nature, and application of cellulose-based hydrogels as an extracellular matrix for tissue growth and regeneration. Hydrogels can be prepared either from native cellulose, including both bacterial and plant sources or from cellulose derivatives, such as methyl cellulose, carboxymethylcellulose, and hydroxypropylmethylcellulose or even metal ions such as silver. Cellulose-polymer composite (polymers that include natural sources including chitosan, starch, alginates, collagen, hyaluronic acid, and chitin) are an attractive, inexpensive, and advantageous structural material that is easy to use. Cellulose-based scaffolding materials are widely used in the regeneration of various tissues, such as bone, cartilage, heart, blood vessel, nerve, and liver, among others. In this review, we discuss the most important applications of cellulosic hydrogels in tissue engineering based on their structural compositions.

**Electronic supplementary material:**

The online version of this article (10.1186/s13036-019-0177-0) contains supplementary material, which is available to authorized users.

## Introduction

Cells communicate with each other either directly via molecular interactions or through the secretion of different hormones or mediators which systematically regulate various cell functions. Growth factors are also secreted during cellular crosstalk and may be pro-proliferative or anti-proliferative in nature, being mainly involved in cell differentiation, migration, adhesion, and gene expression. Natural and synthetic materials may be used as bulking agents for the binding of various growth factors by mimicking natural extracellular matrix (ECM) molecular self-assembly via secondary forces, such as ionic or hydrogen bonds, whereas chemical gels are result of covalent bonds [1–5].

Hydrogels have potential applications in various fields such as agriculture, food, biomaterials, water purification, biomedicine, and pharmaceuticals, among others. [6–8]. Hydrogels are primarily made up of natural living tissue rather than synthetic biomaterials, as a result have a high water content and a soft consistency similar to natural tissues [9]. Moreover, the high water content of these materials contributes to their biocompatibility. Thus, hydrogels can be used as membranes for biosensors [10, 11], in artificial heart and skin [12, 13], contact lenses [14, 15], and drug delivery [3, 6, 16]. Cross-linking synthetic polymer-based hydrogels have been reported, including poly (ethylene glycol) [17, 18], poly (vinyl alcohol) [18, 19], poly (amido-amine) [20], poly (N-isopropylacrylamide) [21], polyacrylamide [18, 22], and poly (acrylic acid) [18, 23].

In tissue engineering, hydrogels are the most extensively used biopolymer due to their highly swollen three- dimensional (3D) environment, which is very similar to soft tissues and allows for the diffusion of nutrients, growth factors and cellular waste through the elastic network and for the regeneration of damaged tissues [13, 18, 24, 25]. In regenerative medicine, hydrogel used to repair and assist regeneration of various soft and hard tissues, such as cartilage, bone and vascular tissues [26–28]. Natural hydrogels include the bioprocessing of natural polymer-based materials such as proteins, including collagen, gelatin, and fibrin, and polysaccharides, including alginate chitosan, hyaluronic acid, dextran, and cellulose which are used as extracellular matrices (ECM).

**Fig. 1:**
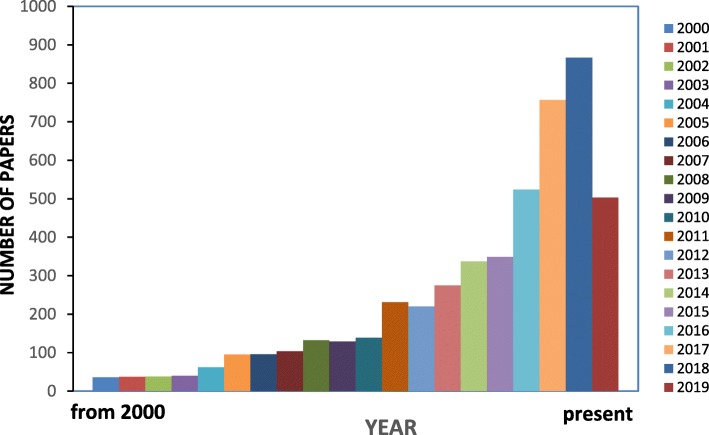
Publications related to cellulose-based hydrogels for tissue engineering (science direct search system; ‘cellulose based hydrogel for tissue engineering’ as search term; https://www.sciencedirect.com)

Cellulose is a fibrous, tough, water-insoluble substance, found in the protective cell walls of plants, particularly in stalks, stems, trunks, and all woody portions. However, it is also produced by some animals (e.g., tunicates), fungi and a few bacteria [29–31]. Due to the presence of abundant hydroxyl groups in the cellulose molecule, cellulose can be used to prepare hydrogels with varying structures and properties to act as a platform for advanced tissue engineering and regenerative medicine. Cellulose-based materials represents a naturally occurring ‘nanomaterial’, and has attracted the attention of researchers all over the world, as shown by the increasing number of annual publications appearing in ‘*Science Direct*’ with ‘cellulose-based hydrogels for tissue engineering’ (Fig. 1) as the search item. However, furthur studies are needed for the development and application of cellulose-based hydrogels. This review highlights the recent development and use of various cellulose-based hydrogels as an ECM and their structural properties for applications in advanced tissue engineering.

## Structure of cellulosic biomass

Cellulose is the most abundant biopolymer and is distributed throughout nature in plants, animals, algae, fungi, and minerals. A major source of cellulose is plant fiber. Cellulose is the main structural component of plants that provides them with their mechanical as well as structural integrity as it contributes approximately 40% to the carbon fraction in plants (Additional file 1: Table S1). Cellulose can be found in its pure form in plants with hemicelluloses, lignins, and other components [32]. Surprisingly, a large fraction of cellulose is produced from trees (wood fiber) with a global production of approximately 1,750,000 kt. Annual plants such as bamboo, cotton linters, jute, flax, sisal, hemp, and ramie also produces significant amount of cellulosic biomass (Additional file 1: Figure S1) [33]. In addition, some fungi and green algae produce cellulose (e.g. *Valonia ventricular*, *Glaucocystis*) and some marine ascidians contain cellulose in their outer cell membrane. Some bacterial genera, such as, *Gluconacetobacter*, *Agrobacterium*, *Pseudomonas*, *Rhizobium*, and *Sarcina* are able to synthesize bacterial cellulose either from glucose or other carbon sources [34–36]. Purified bacterial cellulose is highly crystalline and possess a high degree of polymerization (DP). One of the crucial features of cellulose is its micro-crystalline structure and its synthesis in nature as individual molecules (linear chain of glucosyl residues) which undergo self-assembly at the site of biosynthesis [37].

### Molecular structure of cellulose

Cellulose mainly consist of D-glucopyranose ring units in a ^4^C_1_ configuration, which exhibits the lowest energy conformation [38]. Each unit is linked by β-1, 4-glycosidic linkage that results in an alternate turning of the cellulose chain axis by 180° [39–41]. Within the cellulose chain, three reactive hydroxyl groups (−OH) exist in each anhydroglucose unit (AGU). The –OH groups of the AGU, the oxygen atoms of the D-glucopyranose ring, and the glycosidic linkage interacts with each other within the chain or another cellulose chain by intermolecular and intramolecular hydrogen bonds [42]. The presence of hydrogen bond provides stability to the cellulose molecule and allows it to be a functionally active biomolecule (Additional file 1: Figure S2).

X-ray diffraction studies revealed the crystalline structure of cellulose, and NMR experiments have confirmed its dimorphic and polymorphic nature [43, 44]. Different polymorphs of cellulose are listed in Table 1. Solid-state ^13^C-NMR was used to identify different polymorphs, denoted as cellulose I_α_ and I_β_. Cellulose I_β_ is naturally occurring in plants, whereas cellulose produced by primitive organisms crystallizes in the I_α_ form [55].

**Table 1:** Polymorphs of cellulose

Source	Cellulose polymorphs	Features	References
*Valonia ventricosa*^1^ (bubble algae) *Acetobacter xylinum* (bacteria) *Microdictyon* (green algae) *Halocynthia* (tunicates)	Cellulose I	Native cellulose, found in nature, interconvertible, stable. Crystalline forms are termed as I_α_ and I_β_. I_α_ considered as primitive type, while higher plants possess I_β_.	Marchessault and Sarko, 1967 [45] [46] [47] [48]
*Halicystis* (green algae)^2^ Mutant strain of *A. xylinum*	Cellulose II	Obtained from cellulose I, interconvertible, also found in nature.	[49] [50] [51] Kuga et al., 1993 [52]
Chemical conversion of *Valonia* cellulose I and cellulose II	Cellulose III	Interconvertible and not found in nature. Two crystalline forms isolated as III_I_ and III_II_ respectively.	[49] [50]
Chemical conversion and heating of cellulose III_I_ and III_II_	Cellulose IV	Interconvertible and not found in nature. Two crystalline forms isolated as IV_I_ and IV_II_ respectively.	[53] [54]

^1^highly crystalline cellulose obtained from *Valonia*

^2^naturally occurring cellulose II

**Fig. 2:**
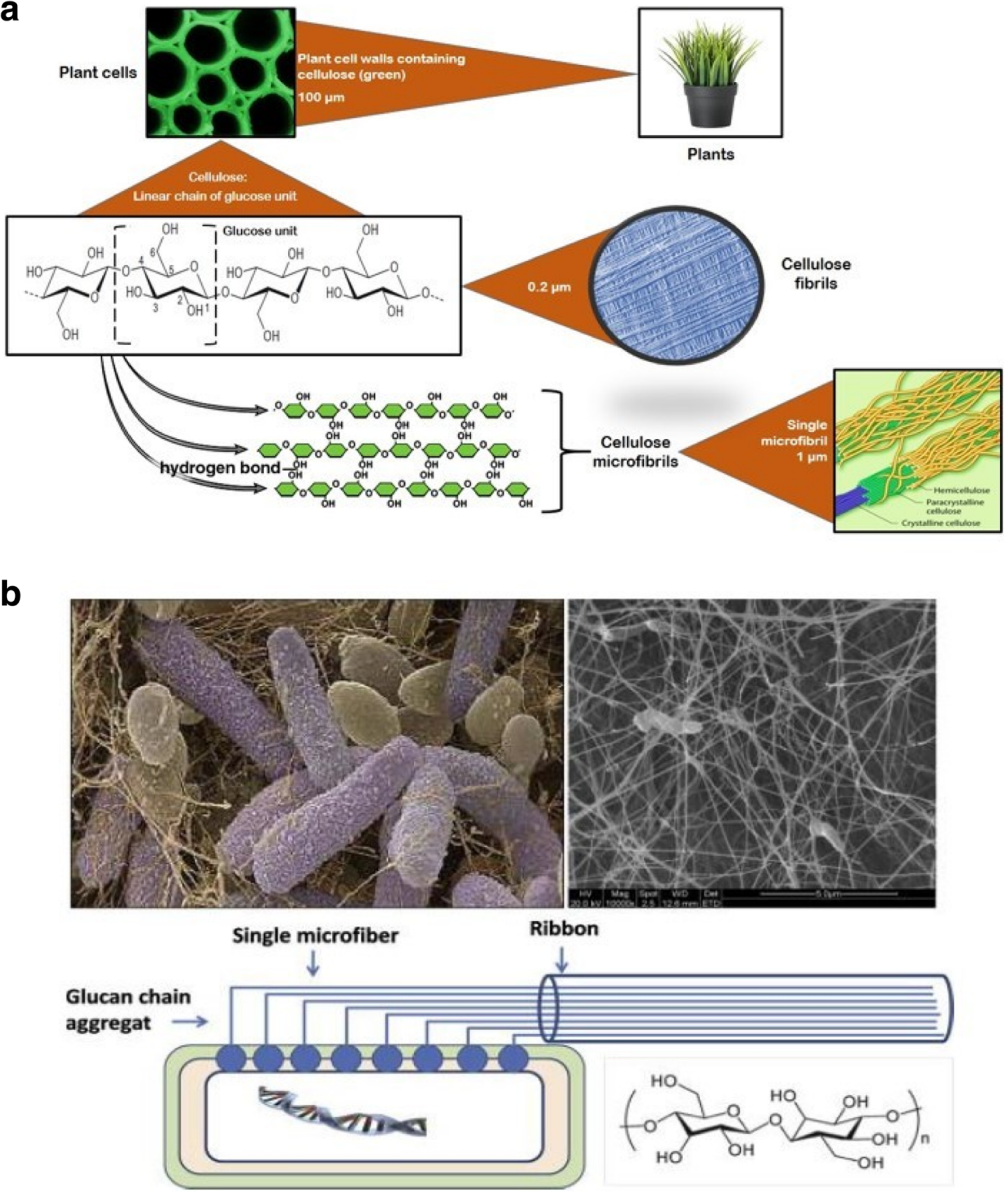
Structure of cellulose and bacterial cellulose. **a** structure of cellulose fibrils (0.2 μm) and microfibrils (1 μm); **b** SEM images of *Acetobacter xylinum* and formation of bacterial cellulose [53] SEM: Scanning electron micrograph

Cellulose chains are arranged in a basic fibrillary unit or elementary fibrils with a length of 0.1 to 0.2 μm and have a characteristic lateral dimension of 0.0015 μm to 0.0035 μm [56, 57]. Such fibrils are known as cellulose fibrils. These fibrils are further assembled into microfibrils with a width of 0.1 μm and a length of 0.1 to 1 μm (Fig. 2a). This fibrillary architecture can be found in both native and man-made fibers [39].

### Structure of plant cellulose (PC)

In the case of plant cell walls, a sheath of amorphous cellulose surrounded by a hemicellulose layer covers the microfibrils [33]. Fibers from different plants vary in morphology and dimension. Additional file 1: Figure S3 clearly shows the variations in the fiber morphologies of cotton (S3a), spruce wood (S3b), and ramie plant (S3c). Surprisingly, all three plants share a common internal structure made up of multiple cell wall layers [58]. During the early growth phase, plant fibers develop a primary cell wall (P layer) that is much thinner than the secondary wall (S layer) formed on its inner side. Inside the S wall, a tertiary cell wall (T layer) is present, which is typically an open, hollow area or lumen-like structure. The cell wall thickness and length of the plant fiber are approximately 4–630 μm and 15–30 μm, respectively. The swelling characteristics as well as their physical and chemical properties are strongly influenced by the configuration, composition, and structure of the P layer, which contains microfibrils crisscrossed onto each other to make a net-like helical structure (S3d-e). The secondary layer is 3–5 μm in thickness and comprises three sublayers (S_1_, S_2_, and S_3_) of which S_2_ is the thickest e (approximately 3–5 μm thickness) as shown in Additional file 1: Figure S3d. The S_2_ layer contains microfibrils arranged in parallel [58–60].

### Structure of bacterial cellulose (BC)

Bacterial cellulose (BC) can be obtained in pure form. Compared to PC, BC contains no hemicellulose or lignin and only a very small amount of carbonyl and carboxyl moieties are present [61]. BC possesses a high degree of crystallinity (above 80%) with a good water retention capacity, and an extraordinary mechanical strength, particularly under wet conditions. One important advantage of using BC is its in-situ molding ability, i.e. shaping during biosynthesis [62]. The culturing and production of BC is the most important part, although it is also important to maintain the pH of the culture medium, since a low pH can often led to the accumulation by-products, such as of gluconic, acetic, or lactic acids [63]. Figure 2b clearly shows the structure and formation of bacterial cellulose in *Acetobacter xylinum*.

## Role of extracellular matrix (ECM)

ECMs are used in tissue engineering and regenerative medicine as a natural model for bioactive modifications. Compared to other ECMs, hydrogels have provided opportunities for the use of a natural ECM as a model for designing biomimetic scaffolds.

### Structure and composition of ECM

The tissues of the human body contain a significant amount of extracellular space, into which ECM molecules are secreted by cells to form a large and complex network. The ECM of the extracellular space provides tissue with mechanical strength, organizes cells into specific tissues, and controls cell behavior and cell differentiation. Two crucial components of the ECM are proteins and glycans, in particular fibrous proteins (e.g., collagen, laminin, and elastin) and glycosaminoglycans (GAGs) [64, 65]. Fibrous proteins act as a scaffold and provide adhesion to matrix structure that are initially embedded in GAGs [65]. Thus, cell-matrix adhesions mediate various physiological responses including cell growth, migration, differentiation, survival, tissue organization and matrix remodeling [66].

### Function of ECM

**Fig. 3:**
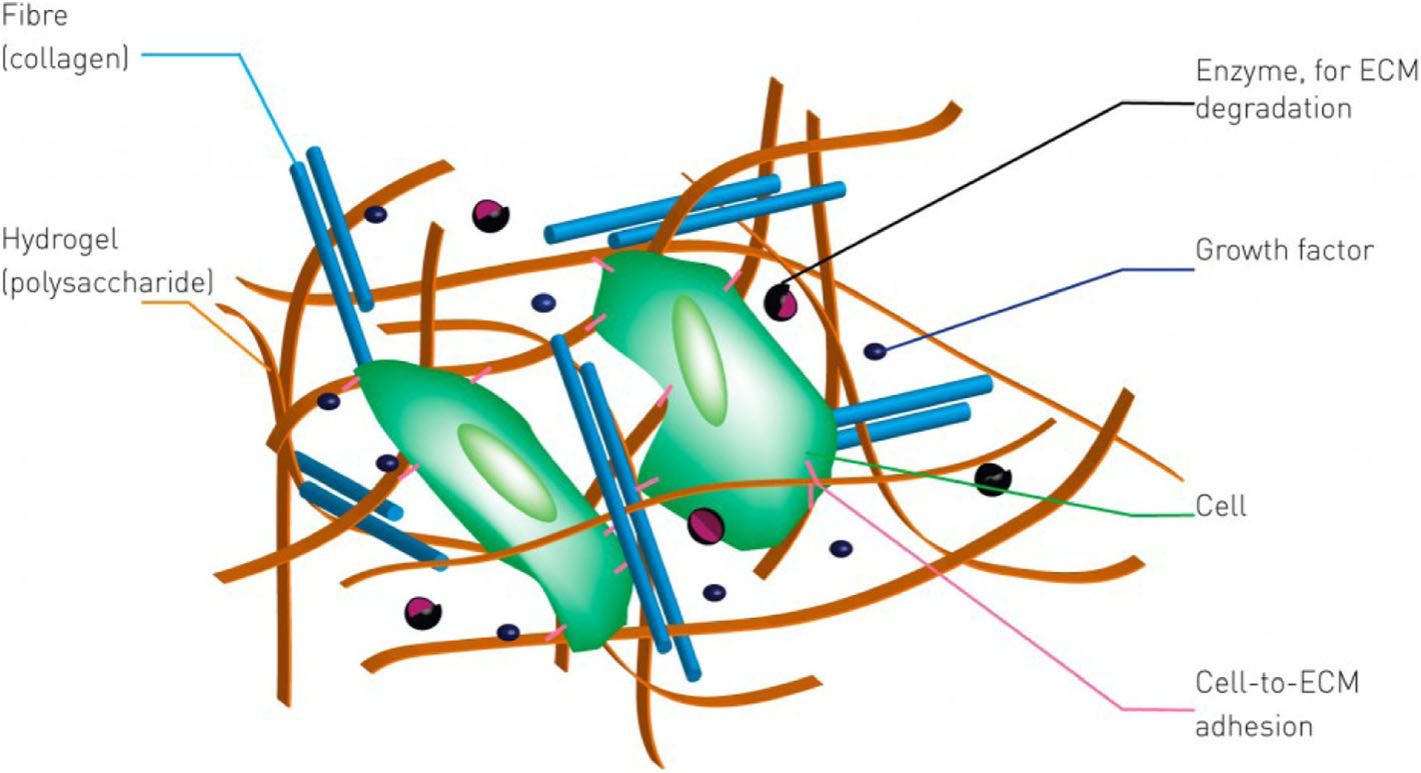
Schematic representation of the extracellular matrix (ECM). In a natural environment, cells (green) use specific markers (pink) to bind to a mechanical support matrix of polysaccharides or hydrogel (yellow) and fibrous proteins (blue). Dissolved proteins like growth factors (purple) enable communication between the cells and matrix-degrading enzymes (black), thus remodeling the matrix [67]

The ECM components undergo self-assembly to form a complex 3D network [18]. Figure 3 shows the role of ECMs in various cellular responses. Cell receptors bind both soluble and tethered signaling cues from the ECM environment. In turn, these receptor-ligand interactions trigger complex cascades of intracellular enzymatic reactions that regulate gene and protein expression and define the fate of a cell in a specific tissue [18, 66]. Cell can also transmits a signal to actively construct and degrade their microenvironment. Thus, the ECMs acts as both a space-filling mechanical scaffold and a bioactive and dynamic environment to mediate cellular functions [64, 65]. However, natural ECMs also provide cellular adhesion, proteolytic degradation and growth factor (GF)- binding [18].

## Basic properties of hydrogels

**Fig. 4:**
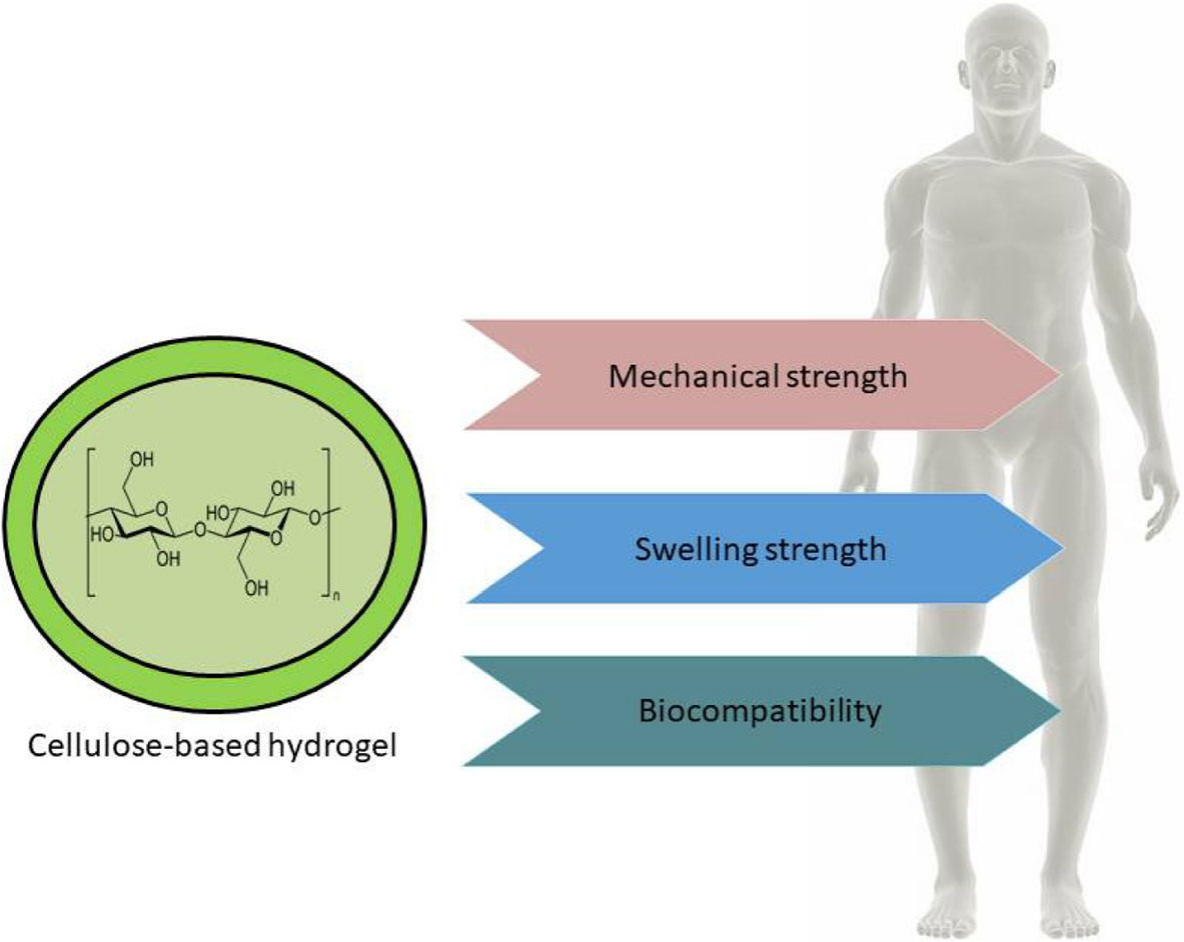
Advantages of the use of cellulose-based hydrogels for tissue engineering

Hydrogels are a type of polymer biomaterials with various properties. In the field of pharmaceutical and biomedical engineering, hydrogels are very important due to their in-vivo swelling properties, mechanical strength and compatibility with biological tissues, facilitating binding (Fig. 4) [68–70].

### Mechanical properties

The mechanical properties of hydrogels are significant from both a pharmaceutical and biomedical point of view [68]. The optimum mechanical strength of a hydrogel is an essential requirement for its successful implementation as a drug delivery system. The excellent mechanical properties of hydrogels allows its physical integrity to be maintained until the cargo molecules are released at a predetermined rate for a predetermined time. The optimum degree of cross-linking may lead to a hydrogel with a suitable mechanical strength. However, by increasing the degree of cross-linking, a stronger form of the hydrogel can be prepared, such as brittle hydrogel that exhibits a decreased percentage of elongation [68, 71].

### Swelling properties

Hydrogels are polymer-based biomaterials developed by the physical or chemical linking of polymers. When hydrogels are exposed to water, they can absorb the water or aqueous fluids without dissolving. This swelling continues until there is an equilibrium between the water and the polymer is established. On the other hand, the elasticity of this biomaterial results from the polymer-polymer interactions that prevent the water flux inside the hydrogel resulting in a state known as “equilibrium swelling” [72].

### Biocompatibility

In the case of tissue engineering and regenerative medicine, hydrogels must be compatible and non-toxic. Biocompatibility is a process that deals with the ability of a hydrogel to perform an appropriate host response in a specific application. Biosafety and bio-functionality are the two keys factors regulating biocompatibility [73]. Polysaccharide-based hydrogels are strikingly important among the polymer hydrogels due to the variety of chemical structures and functional properties [74, 75]. Hydrogels also act as reversible gels with enlargements, such as ionic, H-bonding, or hydrophobic forces which play a crucial role in forming the network [76–78]. The extensive use of hydrogels in the biomedical field is a direct result of their capacity to hold high amount of water, elasticity, biocompatibility, and non-toxicity, among others. The swelling properties of hydrogels results from the presence of hydrophilic groups, such as, −OH, −COOH, −CONH_2_, and -SO_3_H in polymer chains [79]. Swelling is a crucial property of hydrogels for use in biomedical applications, such as in wound dressings [80].

## Cellulose-based hydrogel production

**Fig. 5:**
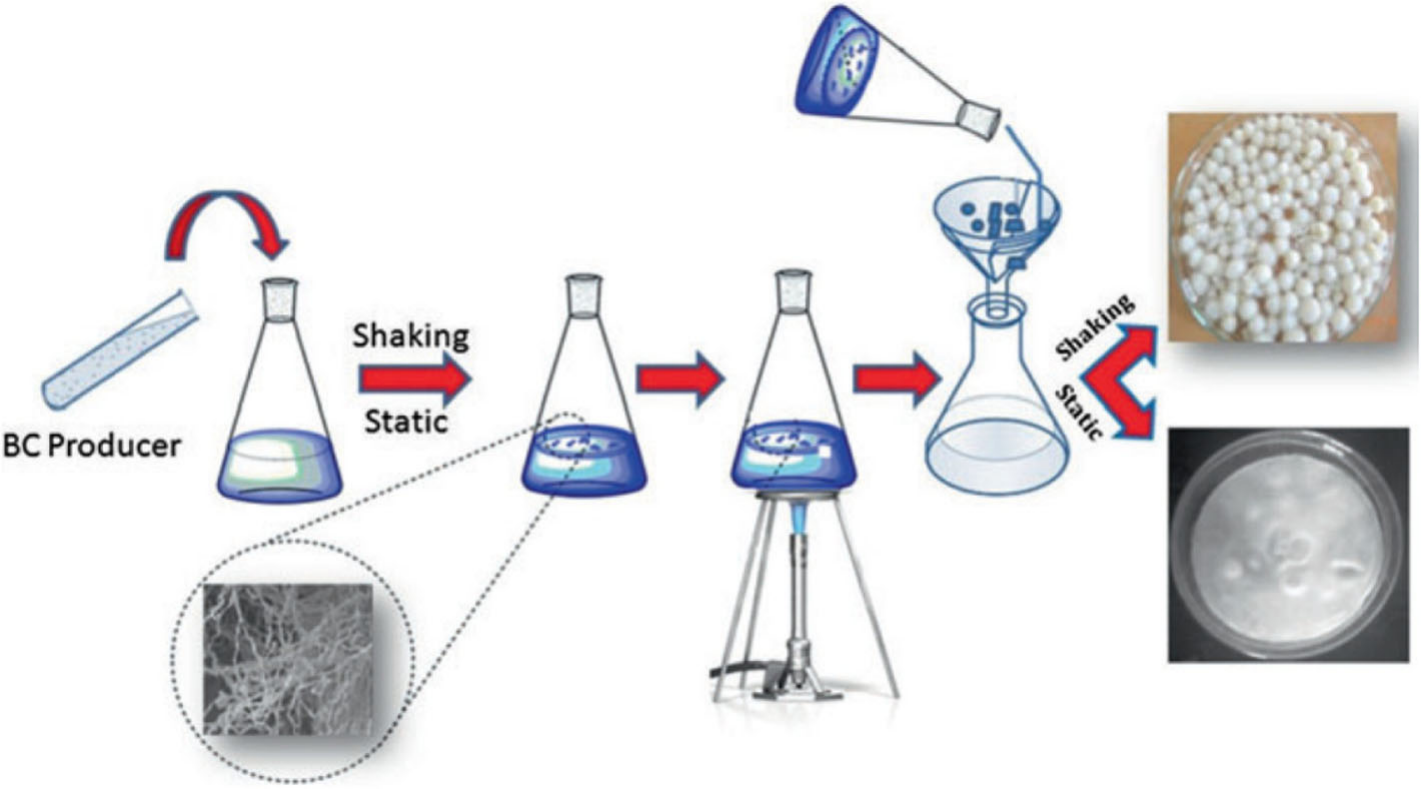
Schematic representation of strategy for BC production [73] BC: bacterial cellulose

The production of cellulose and cellulose-based hydrogel has many advantages in the biomedical and pharmaceutical industries [76]. In addition to plant cellulose (PC) production, microbial cellulose (MC; also known as bacterial cellulose or BC) production is of great importance and is normally carried out using Gram-negative bacteria, such as *Acetobacter xylinum* [81]. Other bacteria used to produces cellulose are listed in Table 2. Bacterial cellulose is produced using either static or shaking culture methods. However, the shaking culture method is more effective than the static culture method; due to the increased growth of bacteria and the high cellulose yield (Fig. 5) [90]. One of the essential features of bacterial cellulose (BC) is the presence of a fine microfibrillar structure that is entirely responsible for its high tensile strength, high crystallinity index, and high degree of polymerization. A previous study found that a hydrogel obtained from BC (0.8%) had a good biocompatibility for use in tissue remodeling[91]. The study also showed the high degree of crystallinity of BC around 89% [92], a high degree of polymerization [93], and a high specific surface area (37 m^2^/g) [94]. Again, BC also showed a large surface area, high aspect ratio, and low bulk density, as well as hydrophilicity [76]. For this reason, BC is widely used in healthcare and medicinal research [95].

**Table 2:** List of some bacteria producing cellulose

Type of bacteria	Example	Application	References
Gram-negative	*Acetobacter xylinum*	Tissue repair material, human tissue substitute or artificial skins; wound dressing	[81]; [82]; [83]; [84]
	*Gluconacetobacter hansenii*	Medical pads, artificial skins	[85]
	*Acetobacter pasteurianus*	Medical pads, membranes	[86]; [87]
	*Rhizobium* sp.	Tissue repair material	[82]; [88]
	*Agrobacterium* sp.	Tissue repair material	[82]; [88]
	*Aerobacter* sp.	Tissue repair material	[88]
	*Azotobacter* sp.	Tissue repair material	[88]
	*Salmonella* sp.	Tissue repair material	[88]
	*Achromobacter* sp.	Tissue repair material	[88]
Gram-positive	*Sarcina ventriculi*	Cell culture, tissue engineering, regenerative medicine	[82]; [88]; [89]

## Processing of cellulose-based hydrogels

Various methods have been employed for the production and processing of hydrogels based on cellulosic materials. Hydrogels can be obtained either directly from native cellulose or from cellulose derivatives [96]. A list of cellulose derivatives, and their solvents, and processing methods is presented in Table 3.

**Table 3:** Summary of some cellulose derivatives and its corresponding hydrogel processing methods

Cellulose/cellulose derivatives	Nature of solvents	Solvent systems	Corresponding hydrogels preparation methods	References
Cellulose form wood	Polar solvents	NMMO	Solution polymerization at 85 °C	[97]
Cellulose from cotton pulp	Polar solvents	LiCl/DMAc	Solution polymerization at 75–90 °C	[98]; [99]; [100]
Filter paper	Ionic solvents	[Amim]Cl	Solution polymerization at 70 °C, 2 h	([101]; [102])
Tunicate cellulose	Alkali aqueous systems	Alkali/urea	Polymerization at −12 to −10 °C, 5–10 min	[103]
Cotton linter	Alkali aqueous systems	Alkali/thiourea	Polymerization at −5 °C, 2–10 min	[104]
Carboxymethylcellulose (CMC)	Polar solvents	H_2_O	Solution polymerization, In situ polymerization	[105]; [106]; [107]
Methyl cellulose (MC)	Polar solvents	DCM/DMSO	Solution polymerization, In situ polymerization	[106]; [108]; [109]
Hydroxyethyl cellulose (HEC)	Polar solvents	H_2_O	Solution polymerization, cryogenic treatment	[106]; [110]
Hydroxypropyl methyl cellulose (HEMP)	Polar solvents	H_2_O/ethanol	Solution polymerization, inverse-phase suspension polymerization	[106]; [111]
Cellulose acetate (CA)	Polar solvents	Acetone/H_2_O	Chemical cross-linking	[112]

*NMMO N*-methylmorpholine-*N*-oxide, *LiCl/DMAc* Lithium chloride/dimethylacetamide, *[Amim] Cl* 1-allyl-3-methylimidazolium chloride, *H*_*2*_*O* water, *DCM/DMSO* Dichloromethane/dimethyl sulfoxide

### Hydrogels obtained from native cellulose

**Fig. 6:**
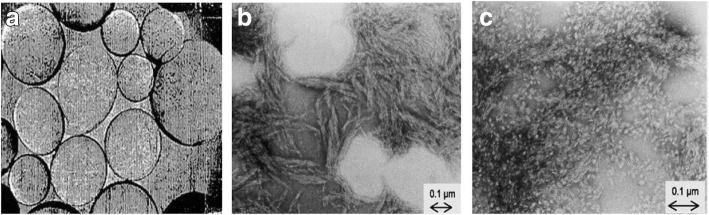
**a** Cellulose hydrogel beads with an average size of 467 μm [99], **b** NMMO fibers, **c** Viscose fibers [115] NMMO: *N*-methylmorpholine-*N*-oxide

A cellulose-based hydrogel can be obtained from a cellulose solution through physical cross-linking. Due to the presence of hydroxyl groups in cellulose, it can easily form cross-linking through hydrogen bonding. The highly extended hydrogen-bonded structure of cellulose results in a compact such that it is not easily dissolved in common solvents [113]. Various solvents have been used to dissolve cellulose. Nowadays, new solvents, such as *N*-methylmorpholine-*N*-oxide (NMMO), ionic liquids (ILs), and alkali/urea (or thiourea) aqueous systems have been developed to dissolve cellulose, with important applications in hydrogel research. However, certain bacterial species are involved in the processing of nearly-pure cellulose hydrogels [96]. Many solvent systems are used to obtain hydrogels from native cellulose. One such systems involves the use of LiCl/DMAc which consists of a mixture of 3 to 15% lithium chloride/LiCl (w/w), dimethylacetamide/DMAc, and 1-methyl-2-pyrrolidinone under specific temperature conditions (normally less than 150 °C) [114]. Cellulose is then dissolved in amide and LiCl in the absence of any polar medium other than amide to obtain hydrogels. However, [99] described the processing of cellulose hydrogels in bead form via the dropwise addition of cellulose solution into DMAc and LiCl to azeotropic methanol or isopropanol as a non-solvent (Fig. 6a). The size of the beaded hydrogels obtained from this method may varies from 100 to 1500 μm [99]. In the LiCl/DMAc system, the cellulose concentration has been determined to be 7 wt%. The presence of water in the cellulose solution is a critical factor for hydrogel production [96]. There have been reports of the rapid dissolution of cellulose at room temperature (around 25 °C) using solvent system with a mixture of dimethylsulfoxide/tertrabutylaluminium fluoride trihydrate (DMSO/TBAF) [116]. Due to its ability to form hydrated dipoles in aqueous solution, TBAF is considered as a suitable solvents for cellulose.

The NMMO solvent system also provides a method for the production of regenerated cellulose fibers, films, food casings, membranes, sponges, and beads, among others without the formation of hazardous byproducts from cellulose solution [115]. Fiber formation occurs in a dry jet-wet spinning process, taking into account several physical factors (e.g. nozzle and air-gap dimensions, drew-down ratio, take-up speed) and dopinge characteristics (cellulose DP and concentration, temperature, modifiers) which influence the shaping process and the final fibers properties. Tertiary amine oxides are also capable of dissolving up to 10% cellulose [117]. A novel method has been developed which produces highly concentrated cellulose, up to 23%, by treating cellulose with NMMO and water [118]. The cellulose fibers generated using the NMMO system are of two types: NMMO fiber and viscose fiber. The NMMO fiber is typically round/oval, homogenous/dense, highly amorphous, and crystalline, as shown in Fig. 6b. On the other hand, viscose fibers are lobate, less homogenous, and more or less amorphous, as indicated in Fig. 6c [119].

Ionic liquids (ILs) also served as a suitable solvent for cellulose and cellulosic materials. Hydrophilic ILs, such as 1-butyl-3-methylimidazolium chloride (BMIMCl) and 1-allyl-3-methylimidazolium chloride (AMIMCl) are commonly used to dissolve cellulose at room temperature (around 25 °C) [120, 121]. After treatment with AMIMCl, regenerated cellulose exhibited excellent mechanical properties. Thus, room temperature ILs represents a new and versatile platform for the comprehensive utilization of cellulose resources and the manufacturing of novel cellulose-based materials with unique properties [121].

Similar to ILs, a cellulose solvent with fast dissolution was developed using a mixture of precooled (− 12 °C) 7 wt% NaOH and 12 wt% urea aqueous solution [[103, 122, 123] in]. Native cellulose dissolved within 2 min in NaOH/urea solution. Thus, this alkali/urea solvent system provides a rapid and convenient method for the rapid-rate dissolution of cellulose.

### Hydrogels obtained from cellulose derivatives

Water-soluble cellulose derivatives are generally biocompatible, and can therefore be used as thickening agents, binding agents, emulsifiers, film formers, suspension aids, surfactants, lubricants, and stabilizers, and in particular as additives in the food, pharmaceutical, and cosmetic industries. Selective cellulose derivatives, including methyl cellulose (MC), hydroxypropyl cellulose (HPC), hydroxypropylmethyl cellulose (HPMC), and carboxymethyl cellulose (CMC) have been used to fabricate cellulose-based hydrogels through physical and chemical cross-linking. In the case of physically cross-linked gels, no covalent bonding formation or breakage takes place, and the cross- linked network is formed through ionic bonding, hydrogen bonding, or an associative polymer- polymer interaction [96]. On the other hand, chemical cross-linked hydrogels are prepared through cross-linking two or more kinds of polymer chains either with a functionalized cross-linker [124] or under UV irradiation [125]. Physically cross-linked hydrogels are widely used in different biomedical fields, including as scaffolds for cell cultures, in cartilage models, and as implants in bone defects [126].

**Fig. 7:**
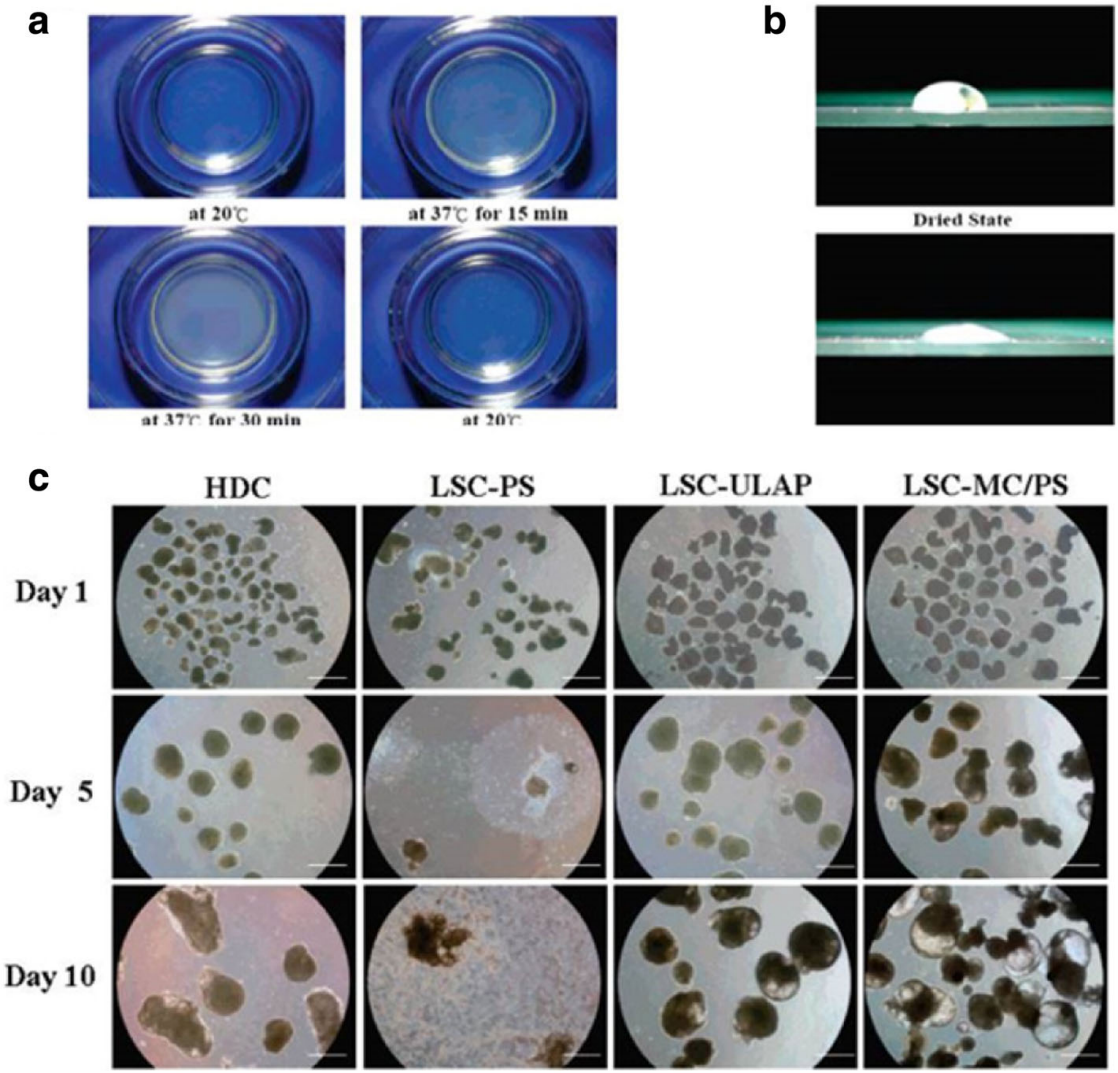
MC-coated hydrogel dishes for hESCs differentiation. **a** Original photograph s of the MC Hydrogel-coated in a polystyrene dish at distinct temperatures; **b** Photograph of a water drop on the surface of the MC hydrogel coated in a polystyrene dish in the dried or hydrated state; **c** Photomicrographs of the hESCs cultivated by different methods for distinct periods (magnification 40x). MC: methyl cellulose; hESCs: human embryonic stem cells; HDC: hanging drop culture; LSC-PS: liquid suspension culture in polystyrene dish; LSC-ULAP: liquid suspension culture in the Corning Ultralow Attachment plate; LSC-MC/PS: liquid suspension culture in the MC-coated polystyrene dish. Scale bars, 1.0 mm [124, 134]

Silated-hydroxypropylmethyl cellulose (Si-HPMC) hydrogels are generally developed for use as scaffold in 3D cultures of osteogenic cells, and are suitable for both in vivo injection and in vitro culturing. However, a previous study presented the use of Si-HPMC hydrogels in osteoblastic survival, proliferation, and differentiation when used as a new scaffold and provided a new treatment technique after bone replacement surgery [127]. MC hydrogels are widely used to mount the surface of polystyrene dishes and are used to cultivate human embryonic stem cells (hESCs) for the formation of embryonic bodies (EBs) in liquid suspension cultures [96, 128, 129]. The EBs developed from the hESCs are shown to express molecular markers specific for representative cells from the three embryonic germ layers, indicating the use of MC-coated dish for the large-scale production of EBs from hESCs as shown in Fig. 7a-c.

### Mixed hydrogels

The mixing or blending of different polymers, such as, a cellulose-polymer composite is a desirable, inexpensive and advantageous method for obtaining novel structural materials [6]. Cellulose (or its derivatives) blended with natural biodegradable polymers, such as chitin, chitosan [130], starch [131, 132], alginates [133, 134], and hyaluronic acid [135], has been used to created novel materials for specific applications. Some examples include the blending of a cellulose-polymer composite with chitosan for the removal of heavy metals, with starch for the food industry, and with alginates for tissue engineering [Chang, 2011].

**Fig. 8:**
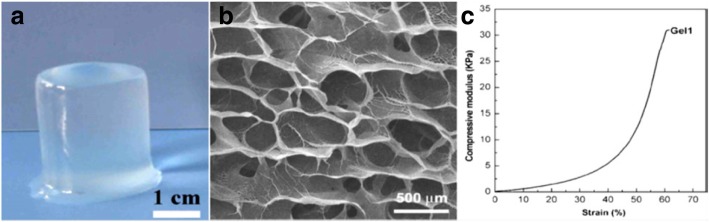
Original photograph (**a**), SEM image (**b**), and compressive stress-strain curve (**c**) of cellulose/SA hydrogel [139] SEM: scanning electron microscopy; SA: sodium alginate

Cellulose-chitosan hydrogel beads areprepared by blending cellulose powder to chitosan solution [136]. Chitosan is perviously blended with a highly concentrated carboxymethylated cellulose solution to form physical hydrogels, which is then cross-linked by irradiation [137]. This cellulose-chitosan duplex has been shown to exert non-diffusible antibacterial properties [128, 129]. A novel microporous hydrogel produced by mixing of cellulose with sodium alginate (SA) solution and then cross linking with epichlorohydrin. The final cellulose/SA hydrogels were characterized by solid-state, ^13^C NMR, wide-angle X-ray diffraction (WXRD), thermo-gravimetric analysis (TGA), scanning electron microscopy (SEM), rheological measurement, dynamic mechanical analysis (DMA), and swelling test analyses to evaluate the structure and morphology of the hydrogels (Fig. 8a-c) [138].

**Fig. 9:**
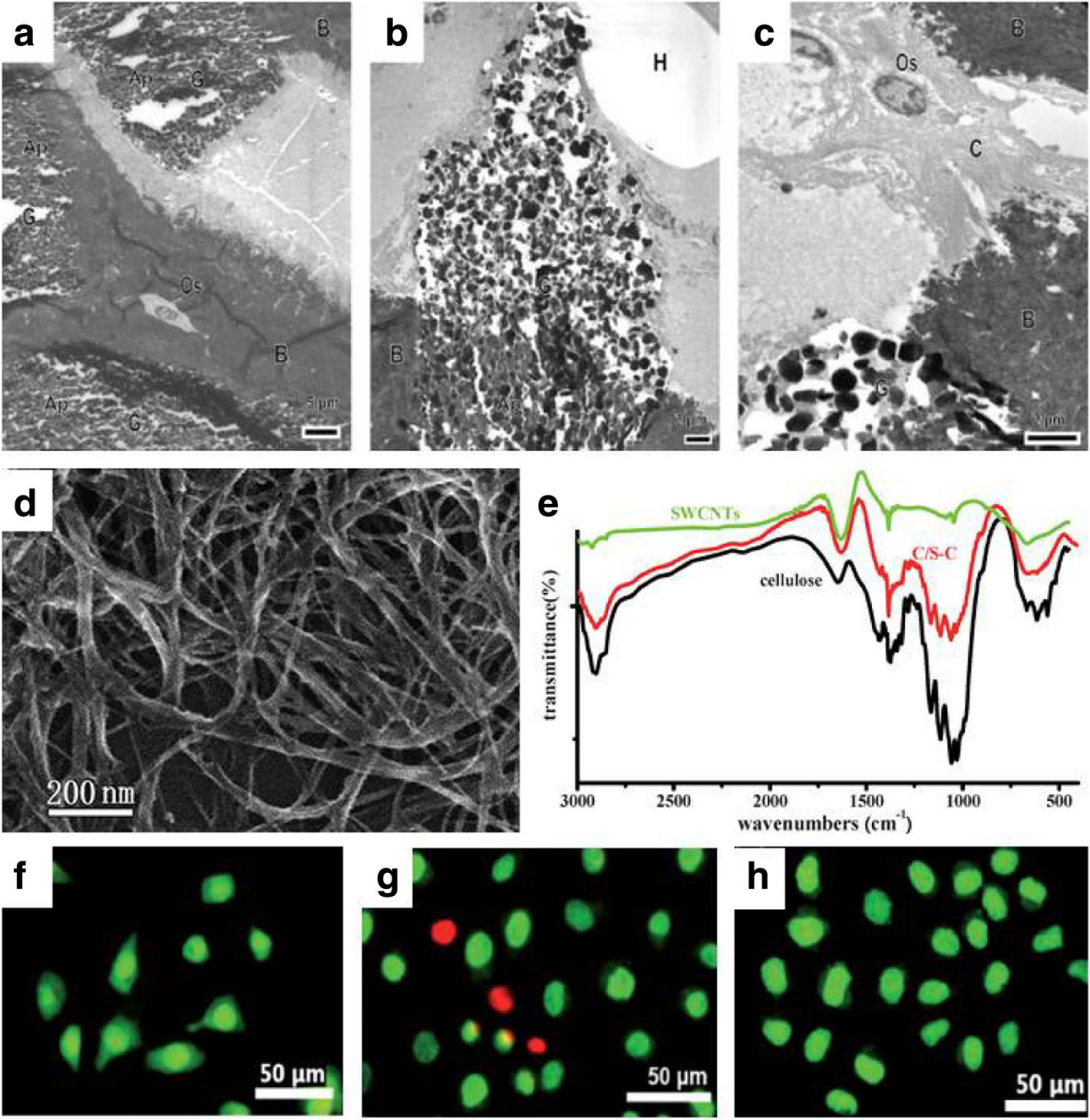
TEM of IBS2-filled bone defects after 8 weeks (a-c). **a** The image clearly showed the mature bone tissue (B) containing the osteocytes (Os); **b** The vacuole containing the Si-HPMC polymer solution (H) around the microporous BPC granules (G) are visible; **c** The precipitation of the biological apatite (Ap) between the BPC crystals, collagen fibers (C), and the nucleus of osteoblastic cells can also be observed. [135]; **d** FE-SEM image of purified SWCNTs; **e** IR spectra of purified SWCNTs, cellulose and C/S-C; f-h. FM images of HeLa cells cultured for 24 h on the C/S-C (**f**), the SWCNTs (**g**), and a glass slide (**h**) [100, 138]. TEM: transmission electron microscopy; Si-HPMC: silated-hydroxypropylmethyl cellulose; BPC: biphasic calcium phosphate; FE-SEM: scanning electron microscopy; SWCNTs: single-walled carbon nanotubes; IR: infrared spectra; C/S-C: cellulose/SWCNTs complex

Currently, polymeric-inorganic hybrid compounds have been widely used in various fields, such as electrical, optical, magnetic, and biological fields, among others [138]. A novel method for the incorporation of inorganic materials and cellulose hydrogels has been studied in New Zealand white rabbits with critically- sized bone defects in the distal femoral epiphyses [139]. In the experimental process, the researchers used an injectable and self-cross-linkable bone substitute (IBS2) composed of Si-HPMC viscous solution (3 wt%) in alkaline medium, supplemented with biphasic calcium phosphate (BCP) ceramic particles. The diameter of the BCP particles ranged from 40 to 80 μm. After a number of weeks, centripetal bone formation was observed near the defects, with a yield strength that was significantly higher than that of the host trabecular bone tissue. Figure 9a-c shows how bone regeneration occurs after the application of Si-HPMC/BCP materials. The use of BC from *Gluconacetobacter hansenii* along with a novel composite material composed of calcium-deficient hydroxyapatite (CdHAP) for orthopedic use has been well characterized and described by [140]. On the other hand, [141] reported the use of heparin/cellulose/charcoal composites to understand the mechanism and crosstalk among cells. To study intracellular drug delivery systems and cellular proliferation, single-walled carbon nanotubes (SWCNTs) wrapped with cellulose have been observed in HeLa cells [101, 102]. Researchers developed SWCNTs with a cellulose solution, dissolved in ionic liquid 1-butyl-3-methylimidazolium bromide (Fig. 9d-e). Another study showed that long cellulose/SWCNT scaffolds could promote the growth of HeLa cells, whereas short cellulose/SWCNT were found to only have a small effect on cell proliferation of HeLa cells (Fig. 9f-h). Healthy cells have a green nucleus, uniform chromatin, and an intact cell membrane, whereas necrotic cells or late apoptotic cells have red nuclei with damaged cell membranes. Cells cultured on a composite scaffold and a glass slide are healthy with a green nucleus (Fig. 9f and h), however, some cells culture on purified SWCNTs are in the late apoptotic stage (Fig. 9g). Thus, inorganic- based cellulosic hydrogels provide a wide range of applications in the biomedical and tissue engineering field.

## Application of cellulose hydrogels in tissue engineering

Cellulose-based hydrogels are used in different fields related to tissue engineering. Patterned macroporous (PM) with a diameter larger than 100 μm were introduced to pristine 3D nanofibrous BC scaffolds using infrared (IR) micromachining techniques to create an in vitro culture model for breast cancer cells (BCs) [142]. PM-BC scaffolds were found to be promote cellular adhesion, growth, proliferation, and infiltration of BCs. *A. xylinum* BC also promotes wound healing as it maintains the wound moist by controlling the wound exudates and also heals severe second-degree burns [143, 144]. Hydroxyethylcellulose (HEC) and carboxymethyl cellulose sodium salt (CMCNa) cross-linked with hyaluronic acid allow for the proliferation of keratinocytes in an in vitro culture [144]. Bacterial nanocellulose (BNC) has great potential for use as a scaffold in tissue engineering, since BC is more effective than PC, which accounts for BC being the first choice in medical and tissue engineering applications.

### BC hydrogels in biomedical applications

BC has promising features due to the similar of its nanostructure and morphology to collagen making BC an attractive choice for use in the support and immobilization of cells. The architecture of BC materials can be engineered at range of scales, ranging from the nano to macroscale by controlling the biofabrication process. BC fibers are solid and, when used in combination with other biocompatible materials, produce nanocomposites particularly suitable for use in human and veterinary medicine [76]. The applications of BC composite hydrogels in biomedicine and tissue engineering are listed in Table 4. BC composites can also be used in cornea formation after cornea surgical treatment, as well as heart and vascular tissue regeneration [148].

**Table 4:** Uses of plant cellulose (PC), microbial cellulose (MC) and bacterial cellulose (BC) composite hydrogels in tissue engineering

Sl. No.	Hydrogel composite	Applications	References
1	Plant cellulose (PC) purified	Tissue engineering and regenerative medicine	[145]; Liu et al., 2014 [146]
2	Algal cellulose (AC)	Bone tissue and cartilage engineering	[147]
3	Bacterial cellulose (BC) purified	Bone tissue engineering, cornea treatment, heart and vascular muscle regeneration	[148]
4	Carboxymethyl cellulose (CMC)	Drug loading and controlled release of drugs, nucleus pulposus	[149]; [148]
5	Polyvinylpyrrolidone (PVP)	Soft-tissue replacement wound management	[149]
6	Gelatin	Wound dressing, tissue regeneration	[80]; [150], [151]
7	Starch	Reinforcement agent for bionanocomposites	[152]
8	Alginate, sodium alginate	High strength hydrogel preparation	[153]
9	Acrylic acid	Burn wound healing	[154]
10	Graphene oxide (GO)	Biomedicine	[155]
11	Vaccarin	Cell growth carrier wound dressing	[156]
12	Hyaluronic acid (HA)	Wound dressing, tissue engineering	[157]
13	Chondroitin sulfate (CS)	Dental material scaffold	Opera et al., 102
14	Calcium phosphate (CP)	Bone substitute	[158]
15	Ca^2+^ activated cellulose, cellulose/lactide	Bone tissue engineering	[148]
16	2-hydroxyethyl methacrylate (PHEMA)	Contact lenses and optic component for biosensors	[159]
17	Polyacrylamide	Cartilage replacement	[160] & [161]
18	Gellan gum	High strength hydrogel for synthetic connective tissue	[153]
19	L-carrageenan	High strength hydrogel for synthetic connective tissue	[153]
20	Hydroxyapatite	Bone scaffold substitute, bone tissue engineering	[162]; [163]; [164]; [165]; [166]
21	Nanohydroxyapatite	Bone tissue engineering	
22	Polyvinyl alcohol (PVA)	Cardiovascular soft tissue replacement, artificial cornea biomaterials	([167]; [168]); [169]; ([170]; [171])
23	Polylacitide and glycidyl methacrylate	Skin repair material	[172]
24	Collagen	Wound dressing for skin regeneration	[173]; [148]
20	Silver	Antimicrobial wound dressing	[174]; [175]

#### Bioactive cartilage implantation

Since, BC gels are free from the action of proteolytic enzymes and reactive oxygen species (ROS), they protects the body from carcinogenesis and prevents the appearance of inflammation. Some examples of cartilage implants composed of BC are septum implants, ear implants, and intervertebral discs, among others [176]. Now a days, the use of bio-mimicking scaffolds has led to the exploration of BC as a potential scaffolding material. A previous study showed that BC did not induce the activation of pro- inflammatory cytokines during in vitro macrophage screening, but rather stimulated the biogenesis of collagen type II with chondrocytes seeded on BC membranes, indicating the suitability of BC as bio-mimicking scaffold [177]. Another more recent study showed the synthesis of modified bacterial cellulose (MBC) from metabolically engineered *Gluconacetobacter xylinus* with a high proliferation level of human mesenchymal stem cells (hMSCs) compared to native cellulose. This material was reported to be a novel in vivo degradable scaffold for chondrogenesis [178, 179].

#### Blood vessel prototypes

Artificial blood vessel-like structures composed of BC are almost 5–25 cm long, which are stable, mechanically strong and resistant to water, aqueous liquids, ions, and small particles, among others. Such vessel-like structure are often used as main platforms for neurotransmitters. Natural BNC has promising mechanical properties, including tear resistance and shape-retention properties, such that it is better suited for use as biological vessels [176].

#### Wound dressing materials

BC has been successfully used as wound dressing material since the 1980s. BC composite materials are used in medicine due to their biocompatible, sterile, porous, and flexible nature. The use of BC sheets allows for wounds to breathe, and prevent the formation of scabs and scars. On the other hand, the use of BC in dressing materials also reduces the amount of pain, protects the skin from infections, and reduces the loss of body fluids. As such, BC composite materials are an ideal candidate for the treatment of wounds and burns [180]. Some examples of commercially available BC composite gels are listed in Table 5. A novel type of BC-based wound dressing, which is impregnated with superoxide dismutase and poviargol, was found to stimulate the healing of thermal skin burns resulting from acute radiation disease [183]. Surprisingly, BC/collagen type I composite was found to promote the reduction of protease, interleukins, and ROS activity in an in vitro culture study [184].

**Table 5:** Commercially available hydrogel wound dressing contains cellulose or its sodium salt. Most dressings are available in two forms, either as sheets or as amorphous gels. Products containing silver ions show antimicrobial property

The hydrogel wound dressing (producer)	Composition	References
IntraSite™ Gel (Smith and Nephew)	Carboxymethycellulose sodium (CMCNa), propylene glycol and water	
GranuGel™ (ConvaTec)	Carboxymethycellulose sodium (CMCNa), Propylene glycol, pectin and water	[181], [182]
Purilon Gel™ (ColoPlast)	Carboxymethycellulose (CMC), calcium alginate and water	
Aquacel Ag™ (ConvaTec)	Carboxymethycellulose sodium (CMCNa) and silver ions (1.2%)	
Silvercel™ (Johnson and Johnson)	Carboxymethycellulose (CMC), silver ions (8%) and calcium alginate	

#### Surgical implants

BCs and BNCs can be used in the form of tracheotomy tubes for reconstructive surgery, such as for artificial heart valves, and as blood vessels in the form of nanotubes or neurotubes for the regeneration of coronary blood vessel and nerves. Previous studies have found new epithelial cell layers to form over these artificial BC tubes, demonstrating the successful application of BC in tissue implantation [185]. The use of PVA/BC nanocomposites for the replacement of cardiovascular tissues has also been reported, since these would mimic the role of natural collagen and elastin (a connective tissue protein that helps skin to return to its original position [167, 168]

#### Potential drug delivery material

Transdermal systems can act as an entry gate for BCs into the domain of drug delivery systems [186]. BC dry films have been obtained after the successful immersion of these in benzalkonium chloride (an antimicrobial agent). Their subsequent drug loading capacity was found to be 0.116 mg/cm^2^ (per unit surface area), and the effect of drug was found to last for at least 24 h against *Staphylococcus aureus* and *Bacillus subtilis* applied to the wounded area [187]. Silver nanoparticle-coated BC fibers showed 99.99% antimicrobial activity against *Escherichia coli* and *S. aureus* [164]. Despite these promising results, the application of BC hydrogels involves certain clinical and pharmacological limitations. However, despite these limitations, the complex nanofibrillar structure of BC represents a suitable macromolecular support for the inclusion of drugs, i.e. for use as a drug carrier [188].

#### Artificial grafting of cornea

Corneal disease is a serious health problem that can lead to partial or complete blindness. An estimated 10 million people have lost their eyesight due to corneal infection or similar diseases. With this in mind, researchers around the world have developed biomaterials for the treatment of defective corneas. The properties of bacterial cellulose, including its nanoporous structure, and excellent mechanical properties, make it an ideal candidate for use as an artificial cornea to help maintain the intraocular pressure of the eye and re-establish ocular pellucidity. The BC/polyvinyl alcohol (BC/PVA) hydrogel has a water content and light transmittance comparable to that of natural cornea and was successfully synthesized and described by Wang et al. for this end.

#### Dental implants

BC composite hydrogels were prepared from *Acetobacter hansenii* by [189] for used in dental root canal treatment (RCT) due to intracanal asepsis. Dental RCT is required when dental caries progress to infection of the dental pulp. From a materials point of view, BC has superior properties compared to plant cellulose (paper points) for the use in dental RCT. Moreover, research has demonstrated the tissue regeneration of periodontal cells after the application of BC hydrogels [190, 191].

### Other applications

Biomimetic scaffolds are of great interest to tissue engineering as they supports essential cell functions. BNC scaffolds in combination with soluble collagen-I stimulate estrogenic differentiation of mesenchymal stem cells (MSCs) [Vielreicher et al., 2018]. The use of cell-derived ECM collagen-I holds good potential, particularly for the tissue engineering of mechanically-challenged tissues. An optimized method for the purification of nano- fibrillated cellulose (NFC) and hydrogel production from wood cellulose was described for the development of a wound dressing material [192]. Inflammation, autolytic debridement, granulated tissue formation, and re- epithelialization are the processes that generally occur during wound healing. Wound dressings are designed to promote healing while protecting the wounds from infection. This is particularly important in cases of chronic wounds (e.g., ulcers), which fail to heal properly. Since a moist environment encourages rapid healing, hydrogels are optimal candidates for the development of wound dressings, either as sheets or in an amorphous form [193]. Various types of hydrogel dressings have been patented so far and are currently commercially available (Table 5), based on synthetic or natural polymers, or a combination of these. Among the most recent patents, it is worth citing those describing in situ forming gels (e.g., based on sprayable formulations [194] and on coalescing nanoparticles [195]), and those exploring radiation crosslinking as a stabilization technique, which allows to obtain sterile and cross-linked hydrogel films in a single-step process [196, 197].

Scaffold attempts to mimic natural ECMs. The most common method of tissue engineering includes the use of biodegradable scaffolds to support the growth and development of cells into tissues or by injecting the isolated single cell suspensions [5]. Cellulose-based scaffolding materials are widely used to regenerate various tissues, such as bone, cartilage, heart, blood vessel, nerve, and liver, among others. However, the design of scaffolds often involves issues related to the need requirement for adequate cell-cell adhesion, cell-cell communication, and cell-ECM communication, which are crucial features of tissue functioning [198]. To overcome these problems, biodegradable scaffolds have been developed. Since, natural polymers are biocompatible, their use allows us to avoid stimulating chronic inflammation or immunological reactions or toxicity. Therefore, hydrogels are used extensively in tissue engineering due to their high swelling properties and their biocompatibility. As a result, they can be incorporated the cells of soft tissues and bioactive molecules via gelling process [199].

## Conclusion and future directions

The current review clearly shows that based explicitly on cellulose biopolymers, hydrogels are a diverse class of materials that have widespread applications in the field of tissue engineering and regenerative medicine. In these areas, scaffolds played a significant role and have been developed to form temporary, artificial ECMs to support cell attachment and three-dimensional (3D) tissue formation. Due to their high mechanical strength and thermostability, bacterial cellulose derivatives are widely used for wound dressing and healing, providing a novel method for the treatment of epidermal burns. Most interestingly, the work of researchers across the globe in the fields of cellulose hydrogel development and characterization seem to indicate that hydrogels based on cellulosic biomaterials could be potential candidates for applications in the field of tissue engineering. However, the research outcomes appear somewhat different from the promising predictions. For example, while using hydrogels in bioengineering applications, researchers have encountered a number of problems. These include difficulties in the handling, maintenance, storage of hydrogels, for example, for hydrogels designed using bioprinters, which are not as much mechanically strong as was theoretically determined. During in vitro experiments it was more difficult to sterilize scaffolding structures than, for example, the cell culture media. Sterilizing by means of autoclaving can cause the functional properties of cellulose-based hydrogels to change. However, their sterilization is necessary since the use of hydrogels without proper sterilization could be a large source of contamination during in vivo and in vitro experiments in laboratory. Researchers have also often encountered difficulties while loading hydrogels with drugs or cells for controlled drug delivery. Further research into hydrogels will be required for the development of new methods and protocols in order to overcome these limitations. Despite these issues, the use of BC hydrogels compared to plant-derived or manmade hydrogels is currently on the rise due to the cost-effective production of BC hydrogels using stirred-tank or static bioreactors. However, more needs to be done to improve plant-derived cellulosic gel production (PC hydrogels). The use of cellulose-based hydrogels in tissue engineering has both advantages and disadvantages, the latter of which will need to be resolved before cellulosic hydrogels can be more widely applied.

Researchers are also working to improve our understanding of the mechanism behind the molecular interaction involved in cellulose ECM materials so that, in the future, materials that mimic natural ECMs in terms of their composition, structural characteristics, and mechanical properties can be developed. The proper development of 3D scaffolding materials could be used to replace conventional tissue engineering techniques to a great extent. Cellulose- based hydrogels have important applications in tissue engineering due to their high biocompatibility and environment- friendly properties. Cellulose-based hydrogels have been recently modified using a nontoxic cross-linking agent or cross-linking treatments, to improve the yield of both the final product and the manufacturing processes. However, further research is needed to develop more advanced cellulose-based hydrogels for use in healthcare and medicine.

## Additional file

Additional file 1: **Table S1.** α**-**Cellulose content of some plant products [197–204]. **Figure S1:** Source of some naturally occurring cellulose. a. hard wood (beech tree); b. cotton tree; c. bamboo; d. *Gluconacetobacter xylinum*; e. ascidians. **Figure S2.** Hydrogen bonding pattern in cellulose molecule. The hydrogen bonding within or between cellulose molecules represents its crystalline nature while studying through X-ray diffraction or NMR technique. **Figure S3.** Microphotograph showing variation in morphology of different fibers. a. twisted cotton fibers; b. tracheids of spruce wood; c. straight fibers of ramie. Copyright permission from [205]; simplified model of plant cell wall. d. structure of S_1_-S_3_ layer; e-f. Cellulose assembly with pectin, hemicellulose, and lignin. Copyright permission from ([49]; [206–208]). (DOCX 312 kb)

## Abbreviations

3D: Three-dimensional
AMIMCl: 1-allyl-3-methylimidazolium chloride
BC: Bacterial cellulose
BCP: Biphasic calcium phosphate
BMIMCl: 1-butyl-3-methylimidazolium chloride
BNC: Bacterial nanocellulose
CdHAP: Calcium-deficient hydroxyapatite
CMC: Carboxymethyl cellulose
CMCNa: Carboxymethylcellulose sodium salt
DMA: Dynamic mechanical analysis
DMAc: Dimethylacetamide
DMSO/TBAF: Dimethysulfoxide/tertrabutylaluminium fluoride trihydrate
DP: Dope characteristics
EBs: Embryonic bodies
ECM: Extracellular matrix
GAGs: Glycosaminglycans
HEC: Hydroxyethylcellulose
hESCs: Human embryonic stem cells
HPC: Hydroxypropyl cellulose
HPMC: Hydroxypropylmethyl cellulose
ILs: Ionic liquids
IR: Infrared
LiCl: Lithium chloride
MBC: Modified bacterial cellulose
MC: Methyl cellulose
MSCs: Mesenchymal stem cells
NaOH: Sodium hydroxide
NFC: Nano-fibrillated cellulose
NMMO: *N*-methylmorpholine-*N*-oxide
NMR: Nuclear magnetic resonance
PC: Plant cellulose
PM: Patterned macroporous
ROS: Reactive oxygen species
SA: Sodium alginate
SEM: Scanning electron microscopy
Si-HPMC: Silated-hydroxypropylmethyl cellulose
SWCNTs: Single-walled carbon nanotubes
TGA: Thermo-gravimetric analysis
UV: Ultra-violet
WXRD: Wide-angle X-ray diffraction
