# Supplementary material for: Functional cellulose-based hydrogels as extracellular matrices for tissue engineering
Source: J Biol Eng. 2019 Jun 20;13:55. doi: 10.1186/s13036-019-0177-0 (PMC6585131; doi:10.1186/s13036-019-0177-0)
Supplement: Supplementary file 1 — Table S1. α-Cellulose content of some plant products [197–204]. Figure S1: Source of some naturally occurring cellulose. a. hard wood (beech tree); b. cotton tree; c. bamboo; d. Gluconacetobacter xylinum; e. ascidians. Figure S2. Hydrogen bonding pattern in cellulose molecule. The hydrogen bonding within or between cellulose molecules represents its crystalline nature while studying through X-ray diffraction or NMR technique. Figure S3. Microphotograph showing variation in morphology of different fibers. a. twisted cotton fibers; b. tracheids of spruce wood; c. straight fibers of ramie. Copyright permission from [205]; simplified model of plant cell wall. d. structure of S1-S3 layer; e-f. Cellulose assembly with pectin, hemicellulose, and lignin. Copyright permission from ([49]; [206–208]). (DOCX 312 kb) [file 13036_2019_177_MOESM1_ESM.docx]

Additional file 1

**Table S1:** α**-**Cellulose content of some plant products.

| Material | α-Cellulose content (%) | References |
| --- | --- | --- |
| *Abies nephrolepis* (khingan fir fibers) | 46 | Chen et al., 2011 |
| *Phyllostachys edulis* (moso bamboo) | 42-86 | Chen et al., 2011; Abe and Yano, 2010 |
| *Triticum aestivum* (wheat fibers) | 40 | Chen et al., 2011 |
| *Linum* sp. (Flax fibers) | 74 | Chen et al., 2011 |
| *Oryza sativa* (rice husk) | 31 | Kumar et al., 2010 |
| *Malus sylvestris* (apple pulp) | 22-34 | Paton, 1974 |
| *Ipomoea batatas* (sweet potato residue) | 84 | Lu et al., 2013 |
| *Gossypium* sp. (cotton fibers) | 97 | De Morais Teixeira et al., 2010 |
| *Andropogon tectorus* (southern gamba grass stem) | 65 |  |
| *Saccharium officinarium* (sugarcane) | 43 | Israel et al., 2008 |
| *Musa paradisiaca* (banana waste) | 37 |  |
| *Zea mays* (maize comb) | 81 |  |
| *Coffea sp.* (Coffee grounds) | 33 | Caetano et al., 2012 |

**
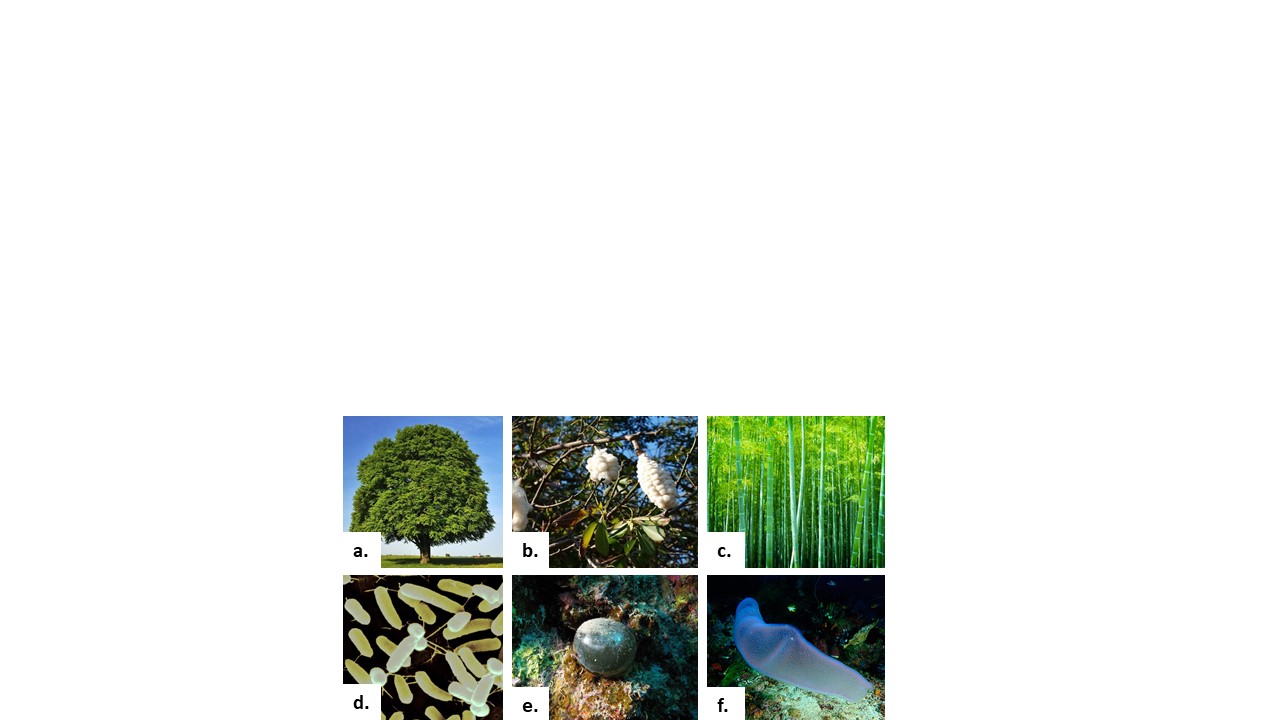
**

**Figure S1:** Source of some naturally occurring cellulose. a. hard wood (beech tree); b. cotton tree; c. bamboo; d. *Gluconacetobacter xylinum*; e. ascidians.


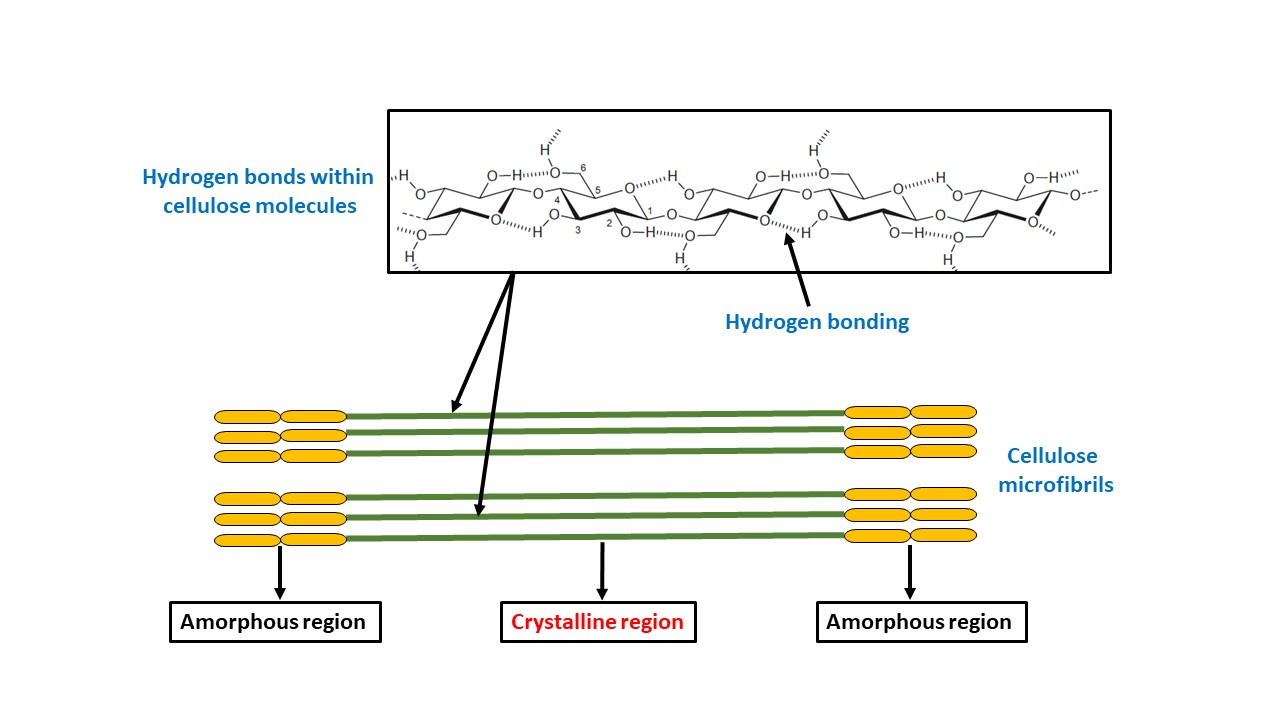


**Figure S2.** Hydrogen bonding pattern in cellulose molecule. The hydrogen bonding within or between cellulose molecules represents its crystalline nature while studying through X-ray diffraction or NMR technique.


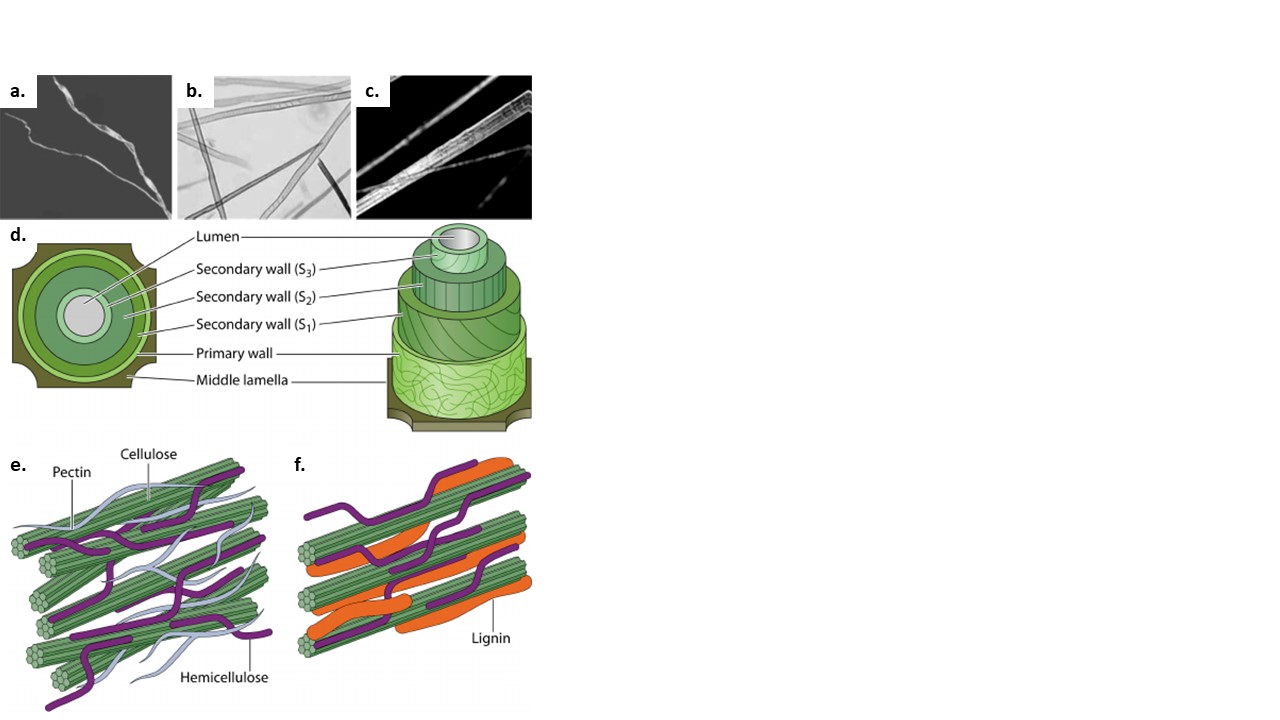


**Figure S3.** Microphotograph showing variation in morphology of different fibers. a. twisted cotton fibers; b. tracheids of spruce wood; c. straight fibers of ramie [Loelovich and Leykin, 2008]; simplified model of plant cell wall. d. structure of S_1_-S_3_ layer; e-f. Cellulose assembly with pectin, hemicellulose, and lignin [Rytioja et al., 2014]

**References:**

Chen W, Yu H, Liu Y, Hai Y, Zhang M, Chen P. Isolation and characterization of cellulose nanofibers from four plant cellulose fibers using a chemical-ultrasound process. Cellulose. 2011; doi: 10.1007%2Fs10570-011-9497-z.

Abe K, Yano H. Comparison of the characteristics of cellulose microfibril aggregates isolated from fiber and parenchyma cells of Moso bamboo (Phyllostachys pubescens). Cellulose. 2010; doi: 10.1007/s10570-009-9382-1.

#### Kumar PS, Ramakrishnan K, Kirupha SD, Sivanesan S. Thermodynamics and kinetic studies of cadmium adsorption from aqueous onto rice husk. Braz J Chem Eng. 2010; doi: 10.1590/S0104-66322010000200013.

Paton D. Cellulose from apple tissue: isolation, purification and chemical modification. Can Inst Food Sci Technol. 1974; 7:61-64.

Lu H, Gui Y, Zheng L, Liu X. Morphological, crystalline, thermal and physicochemical properties of cellulose nanocrystals obtained from sweet potato residue. Food Res Int. 2013; doi: [10.1016/j.foodres.2012.10.013](https://doi.org/10.1016/j.foodres.2012.10.013).

De Morais Teixeira E, Correa AN, Manzoli A, de Lima Leite F, de Oliveria CR. Cellulose nanofibers from white and naturally colored cotton fibers. Cellulose. 2010; doi: 10.1007/s10570-010-9403-0.

Israel AU, Obot IB, Umoren SA, Mkpenie V, Asuquo JE. Production of cellulosic polymers from agricultural waste. E-J Chem. 2008; 5:81-85.

Caetano NS, Silva VFM, Mata, TM. Valorization of coffee grounds for biodiesel production. Chem Eng Trans. 2012; doi: 10.3303/CET1226045.

Loelovich M, Leykin A. Structural investigation of various cotton fibers and cotton celluloses. BioResources. 2008; 3:170-177.

Rytioja J, Hilden K, Yuzon J, Hatakka A, de Vries RP, Makela MR. Plant-polysaccharide-degrading enzymes from basidiomycetes. Microbiol Mol Biol Rev. 2014; doi: 10.1128/MMBR.00035-14.
